# Supplementary material for: Pre-Clinical Investigations of the Pharmacodynamics of Immunogenic Smart Radiotherapy Biomaterials (iSRB)
Source: Pharmaceutics. 2023 Dec 14;15(12):2778. doi: 10.3390/pharmaceutics15122778 (PMC10747552; doi:10.3390/pharmaceutics15122778)
Supplement: Supplementary file 1 [file pharmaceutics-15-02778-s001.zip › Supplementary Materials updated.pdf]

Pre-clinical Investigations of the Pharmacodynamics of Immunogenic Smart Radiotherapy Biomaterials (iSRB)

Michele Moreau [1,2,3,*], Shahinur Acter [2], Lindokuhle Ngema [2,4], Noella Bih [1], Gnagna Sy [2], Lensa S. Keno [5], Kwok-Fan Chow [3], Erno Sajo [3], Oscar Nebangwa [6], Jacques Walker [6], Philmo Oh [6], Eric Broyles [6], Wilfred Ngwa [1,2,3,*] and Sayeda Yasmin-Karim [1]

Supplementary Materials

S1. Mice's Body Score.

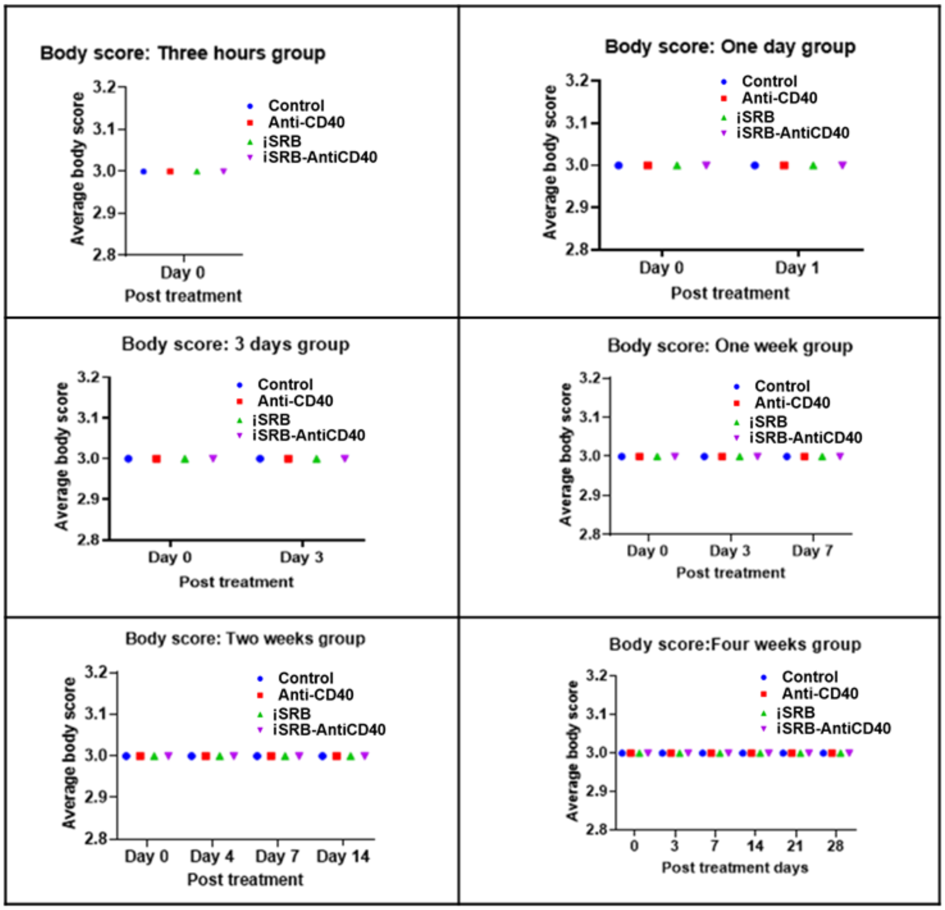

Figure S1. Mice body scores for time points of 3 hours, days 1, 3, 7, 14, and 30 post-treatments. There were no variations in the mice's body scores for all the above collections (n=3/cohort/post-treatment day).

Table S1.

Hepatic and Renal Function Panels in male mice at different times post-treatment (data presented as mean and standard deviation (SD), n = 3)							
**Time point**	**Key Parameters**	**Male**					
		**No Treatment**		**iSRB**		**iSRB_Anti-CD40**	
		**Mean**	**SD**	**Mean**	**SD**	**Mean**	**SD**
**Day 1**	**Alkaline Phosphatase (9.60 - 218.85 U/L)**	43.67	7.09	42.33	7.02	52.00	6.00
	**Calcium (8.28 - 12.27 mg/dL)**	7.80	0.46	7.53	1.63	9.17	0.49
	**Cholesterol (52.62 - 167.47 mg/dL)**	100.33	7.57	113.00	7.00	96.67	2.52
	**Glucose (106.74 - 353.74 mg/dL)**	243.00	31.10	308.00	42.30	269.00	45.71
	**Triglycerides (11.26 - 254.54 mg/dL)**	107.00	13.00	87.33	18.01	91.00	7.94
	**Phosphorus (3.61 - 13.19mg/dL)**	10.70	2.10	12.97	3.32	9.13	3.11
	**Albumin (1.92 - 4.11 g/dL)**	3.20	0.44	3.03	0.21	2.70	0.00
	**Globulin (0.92-3.64 g/dL)**	2.07	0.40	2.50	0.36	1.97	0.06
	**Sodium (124.81 - 160.31 mmol/L)**	148.17	1.85	149.43	1.75	136.83	18.22
	**Potassium (2.17 - 8.18 mmol/L)**	5.68	0.14	5.83	0.24	5.48	0.16
	**Chloride (99.96 - 121.75 mmol/L)**	118.50	1.73	119.80	3.27	118.33	4.01
	**Bicarbonate (5.86 - 30.19 mEq/L)**	19.50	2.98	22.87	1.69	13.93	1.27
	**ALB/GLOB ratio**	1.61	0.51	1.23	0.20	1.37	0.04
	**BUN/Creatinine ratio**	84.69	33.94	70.33	23.32	80.23	27.84
	**Na/K ratio**	26.08	0.32	25.65	1.32	24.97	3.30
	**Total Protein (4.48 - 6.32 g/dL)**	5.27	0.32	5.53	0.42	4.67	0.06
**Day 14**	**Alkaline Phosphatase (9.60 - 218.85 U/L)**	57.33	10.41	51.33	4.93	54.00	7.21
	**Calcium (8.28 - 12.27 mg/dL)**	9.90	0.30	10.13	0.17	9.60	0.26
	**Cholesterol (52.62 - 167.47 mg/dL)**	93.33	8.02	76.00	4.58	75.67	9.02
	**Glucose (106.74 - 353.74 mg/dL)**	294.00	28.83	297.67	24.79	319.00	42.51
	**Triglycerides (11.26 - 254.54 mg/dL)**	102.33	10.26	77.00	5.69	85.00	9.54
	**Phosphorus (3.61 - 13.19mg/dL)**	8.70	0.50	9.77	0.40	9.90	0.26
	**Albumin (1.92 - 4.11 g/dL)**	2.73	0.12	2.73	0.17	2.93	0.06
	**Globulin (0.92-3.64 g/dL)**	1.80	0.26	2.27	0.10	2.20	0.20
	**Sodium (124.81 - 160.31 mmol/L)**	151.37	3.51	149.50	0.85	152.40	4.49
	**Potassium (2.17 - 8.18 mmol/L)**	5.10	0.32	4.94	0.14	5.10	0.13
	**Chloride (99.96 - 121.75 mmol/L)**	118.17	2.48	116.00	2.17	118.57	1.10
	**Bicarbonate (5.86 - 30.19 mEq/L)**	12.33	1.90	10.30	0.30	14.93	0.40
	**ALB/GLOB ratio**	1.94	0.28	1.50	0.11	1.34	0.15
	**BUN/Creatinine ratio**	68.72	6.34	100.32	10.48	80.14	4.47
	**Na/K ratio**	30.73	1.36	30.24	0.84	29.90	0.27
	**Total Protein (4.48 - 6.32 g/dL)**	4.53	0.25	5.00	0.17	5.13	0.15
**Day 30**	**Alkaline Phosphatase (9.60 - 218.85 U/L)**	61.00	15.39	68.33	0.58	65.67	4.04
	**Calcium (8.28 - 12.27 mg/dL)**	8.10	1.42	9.60	0.10	8.37	1.00
	**Cholesterol (52.62 - 167.47 mg/dL)**	98.33	4.16	102.00	7.55	107.33	5.03
	**Glucose (106.74 - 353.74 mg/dL)**	270.33	15.95	307.33	14.01	287.67	15.01

	**Triglycerides (11.26 - 254.54 mg/dL)**	129.00	14.18	137.67	8.50	146.00	9.54
	**Phosphorus (3.61 - 13.19mg/dL)**	7.73	1.18	7.13	0.38	8.53	0.95
	**Albumin (1.92 - 4.11 g/dL)**	3.03	0.25	2.97	0.06	3.30	0.10
	**Globulin (0.92-3.64 g/dL)**	2.13	0.32	2.20	0.10	2.33	0.15
	**Sodium (124.81 - 160.31 mmol/L)**	150.40	4.30	146.60	1.97	145.90	2.10
	**Potassium (2.17 - 8.18 mmol/L)**	5.21	0.27	5.03	0.43	4.89	0.22
	**Chloride (99.96 - 121.75 mmol/L)**	119.20	4.45	119.40	3.60	117.67	3.01
	**Bicarbonate (5.86 - 30.19 mEq/L)**	21.83	2.93	21.17	1.78	24.57	1.91
	**ALB/GLOB ratio**	1.43	0.10	1.35	0.04	1.42	0.07
	**BUN/Creatinine ratio**	112.42	27.73	116.14	20.27	103.22	20.00
	**Na/K ratio**	28.88	1.04	29.27	2.26	29.86	1.28
	**Total Protein (4.48 - 6.32 g/dL)**	5.17	0.57	5.03	0.15	5.63	0.22
**Day 90**	**Alkaline Phosphatase (9.60 - 218.85 U/L)**	44.00	7.55	35.00	2.65	54.00	7.94
	**Calcium (8.28 - 12.27 mg/dL)**	8.07	0.21	7.27	0.15	8.67	0.45
	**Cholesterol (52.62 - 167.47 mg/dL)**	111.67	13.65	90.67	11.24	113.33	4.16
	**Glucose (106.74 - 353.74 mg/dL)**	301.33	17.62	255.67	30.37	363.00	32.42
	**Triglycerides (11.26 - 254.54 mg/dL)**	136.00	8.19	123.33	11.37	134.33	8.50
	**Phosphorus (3.61 - 13.19mg/dL)**	9.97	0.25	10.57	0.81	8.73	0.25
	**Albumin (1.92 - 4.11 g/dL)**	3.30	0.17	3.33	0.06	3.33	0.21
	**Globulin (0.92-3.64 g/dL)**	2.60	0.10	2.80	0.10	2.63	0.45
	**Sodium (124.81 - 160.31 mmol/L)**	149.03	2.06	147.47	3.16	149.43	1.80
	**Potassium (2.17 - 8.18 mmol/L)**	5.17	0.27	4.92	0.19	5.22	0.22
	**Chloride (99.96 - 121.75 mmol/L)**	120.00	2.75	117.50	0.75	118.90	3.12
	**Bicarbonate (5.86 - 30.19 mEq/L)**	8.50	0.10	7.53	0.25	10.17	0.38
	**ALB/GLOB ratio**	1.27	0.11	1.19	0.06	1.30	0.30
	**BUN/Creatinine ratio**	113.70	8.39	107.88	6.43	131.61	23.50
	**Na/K ratio**	28.87	1.22	30.01	1.43	28.64	0.90
	**Total Protein (4.48 - 6.32 g/dL)**	5.90	0.10	6.13	0.06	5.97	0.25

Table S1. Male mice Hepatic and Renal function parameters following cardiocentesis blood sampling corresponding to days 1, 14, 30, and 90 post-treatment with the annotated reference range for each parameter noted in the table.

Table S2.

Hepatic and Renal Function Panels in female mice at different times post-treatment (data presented as mean and standard deviation (SD), n = 3)							
**Time point**	**Key Parameters**	**Female**					
		**No Treatment**		**iSRB**		**iSRB_Anti-CD40**	
		**Mean**	**SD**	**Mean**	**SD**	**Mean**	**SD**
**Day 1**	**Alkaline Phosphatase (9.60 - 218.85 U/L)**	83.67	17.62	73.33	9.07	65.00	3.61
	**Calcium (8.28 - 12.27 mg/dL)**	7.67	1.69	8.30	1.42	6.77	1.76
	**Cholesterol (52.62 - 167.47 mg/dL)**	94.33	4.93	88.33	10.21	75.33	14.22
	**Glucose (106.74 - 353.74 mg/dL)**	274.67	49.22	276.67	10.69	230.00	43.35
	**Triglycerides (11.26 - 254.54 mg/dL)**	97.00	37.27	85.67	23.71	89.00	7.94
	**Phosphorus (3.61 - 13.19mg/dL)**	10.97	1.00	9.47	1.55	11.40	1.04
	**Albumin (1.92 - 4.11 g/dL)**	2.93	0.06	3.03	0.06	2.70	0.35
	**Globulin (0.92-3.64 g/dL)**	2.50	0.36	1.77	0.21	2.33	0.40
	**Sodium (124.81 - 160.31 mmol/L)**	152.07	2.24	148.90	3.49	149.23	1.58
	**Potassium (2.17 - 8.18 mmol/L)**	5.76	0.24	5.78	0.13	5.87	0.16
	**Chloride (99.96 - 121.75 mmol/L)**	118.40	0.75	118.73	2.91	119.23	0.75
	**Bicarbonate (5.86 - 30.19 mEq/L)**	15.30	1.50	15.30	4.01	18.13	2.87
	**ALB/GLOB ratio**	1.19	0.17	1.73	0.20	1.20	0.34
	**BUN/Creatinine ratio**	78.07	13.06	112.23	36.89	112.23	11.82
	**Na/K ratio**	26.44	0.83	25.76	0.56	25.76	0.54
	**Total Protein (4.48 - 6.32 g/dL)**	5.43	0.35	4.80	0.20	5.03	0.25
**Day 14**	**Alkaline Phosphatase (9.60 - 218.85 U/L)**	115.33	8.50	118.67	4.93	110.67	16.26
	**Calcium (8.28 - 12.27 mg/dL)**	9.33	0.35	9.60	0.17	9.87	0.31
	**Cholesterol (52.62 - 167.47 mg/dL)**	70.67	3.06	76.00	4.58	75.67	4.51
	**Glucose (106.74 - 353.74 mg/dL)**	310.67	28.54	211.33	24.79	223.67	31.21
	**Triglycerides (11.26 - 254.54 mg/dL)**	59.33	9.07	75.33	5.69	80.33	9.07
	**Phosphorus (3.61 - 13.19mg/dL)**	9.47	0.15	10.13	0.40	8.23	0.15
	**Albumin (1.92 - 4.11 g/dL)**	3.03	0.15	3.00	0.17	3.13	0.15
	**Globulin (0.92-3.64 g/dL)**	1.57	0.30	2.00	0.10	2.00	0.30
	**Sodium (124.81 - 160.31 mmol/L)**	151.20	3.09	152.33	0.85	151.17	3.09
	**Potassium (2.17 - 8.18 mmol/L)**	4.93	0.08	5.04	0.14	5.15	0.08
	**Chloride (99.96 - 121.75 mmol/L)**	118.17	2.27	116.00	2.17	116.93	2.27
	**Bicarbonate (5.86 - 30.19 mEq/L)**	12.33	1.20	10.30	0.30	11.93	1.20
	**ALB/GLOB ratio**	1.94	0.32	1.50	0.11	1.60	0.32
	**BUN/Creatinine ratio**	68.72	4.17	100.32	10.48	82.50	4.17
	**Na/K ratio**	30.73	1.01	30.24	0.84	29.36	1.01
	**Total Protein (4.48 - 6.32 g/dL)**	4.60	0.20	5.00	0.20	5.13	0.15
**Day 30**	**Alkaline Phosphatase (9.60 - 218.85 U/L)**	95.33	5.13	91.33	9.71	95.00	22.91
	**Calcium (8.28 - 12.27 mg/dL)**	8.23	0.93	7.13	0.70	8.17	0.93

	**Cholesterol (52.62 - 167.47 mg/dL)**	91.33	7.77	78.33	15.89	82.67	7.51
	**Glucose (106.74 - 353.74 mg/dL)**	278.33	31.09	258.00	46.70	268.00	10.15
	**Triglycerides (11.26 - 254.54 mg/dL)**	138.33	21.39	131.33	12.66	105.33	15.14
	**Phosphorus (3.61 - 13.19mg/dL)**	7.17	0.21	7.10	0.35	6.47	0.90
	**Albumin (1.92 - 4.11 g/dL)**	3.20	0.10	2.90	0.61	2.97	0.06
	**Globulin (0.92-3.64 g/dL)**	2.23	0.15	2.40	0.36	1.93	0.12
	**Sodium (124.81 - 160.31 mmol/L)**	152.07	3.10	139.87	19.57	153.03	2.11
	**Potassium (2.17 - 8.18 mmol/L)**	4.99	0.12	5.46	0.15	5.54	0.24
	**Chloride (99.96 - 121.75 mmol/L)**	118.17	2.31	120.27	2.32	119.70	2.91
	**Bicarbonate (5.86 - 30.19 mEq/L)**	19.47	1.36	24.73	2.40	22.70	3.24
	**ALB/GLOB ratio**	1.44	0.05	1.25	0.40	1.54	0.12
	**BUN/Creatinine ratio**	65.12	18.05	52.69	0.23	69.61	23.84
	**Na/K ratio**	30.47	1.26	25.57	3.06	27.66	0.83
	**Total Protein (4.48 - 6.32 g/dL)**	5.43	0.25	5.30	0.30	4.90	0.24
**Day 90**	**Alkaline Phosphatase (9.60 - 218.85 U/L)**	59.67	2.52	59.00	8.89	34.00	3.46
	**Calcium (8.28 - 12.27 mg/dL)**	7.60	0.17	7.80	0.26	7.07	0.25
	**Cholesterol (52.62 - 167.47 mg/dL)**	87.67	5.69	86.00	7.55	89.33	5.86
	**Glucose (106.74 - 353.74 mg/dL)**	330.33	27.30	318.67	17.04	288.67	16.86
	**Triglycerides (11.26 - 254.54 mg/dL)**	106.67	8.02	95.33	7.02	103.67	6.43
	**Phosphorus (3.61 - 13.19mg/dL)**	10.27	0.21	7.50	0.17	9.20	0.10
	**Albumin (1.92 - 4.11 g/dL)**	3.33	0.15	3.27	0.15	2.47	0.12
	**Globulin (0.92-3.64 g/dL)**	2.30	0.17	2.40	0.10	3.07	0.15
	**Sodium (124.81 - 160.31 mmol/L)**	152.83	1.25	151.77	3.46	149.30	1.18
	**Potassium (2.17 - 8.18 mmol/L)**	5.14	0.25	5.19	0.11	4.93	0.32
	**Chloride (99.96 - 121.75 mmol/L)**	120.80	1.71	118.93	2.60	118.20	3.42
	**Bicarbonate (5.86 - 30.19 mEq/L)**	8.23	0.32	9.63	0.29	6.77	0.38
	**ALB/GLOB ratio**	1.46	0.18	1.36	0.10	0.81	0.08
	**BUN/Creatinine ratio**	100.35	15.25	107.95	6.97	53.66	12.97
	**Na/K ratio**	29.77	1.22	29.23	0.48	30.38	2.23
	**Total Protein (4.48 - 6.32 g/dL)**	5.63	0.06	5.67	0.15	5.53	0.32

Table S2. Following the Cardiocentesis blood sampling method, female mice have Hepatic and Renal function parameters corresponding to days 1, 14, 30, and 90 post-treatment with the annotated reference range for each parameter as noted in the table.

Table S3. Hematology analysis from mice (males vs. females) at different time points post-treatment (data presented as mean ± SD, n = 3)

Time point	Key parameter(s)	Male			Female		
		iSRB_Ab-CD40	iSRB	No treatment	iSRB_Ab-CD40	iSRB	No treatment
3 hrs	**WBC** (3.20 – 12.70 K/uL)	5.75 ± 0.64	-	-	4.32 ± 0.42	-	-
	**RBC** (7.00 – 10.10 M/uL)	8.98 ± 0.28	-	-	9.90 ± 0.13	-	-
	**HGB** (11.80 – 14.90 g/dL)	13.65 ± 0.21	-	-	14.95 ± 0.49	-	-
	**MCH** (13.80 – 18.40 pg)	15.20 ± 0.28	-	-	15.10 ± 0.28	-	-
	**MCHC** (31.00 – 34.70 g/dL)	31.60 ± 0.57	-	-	32.15 ± 1.48	-	-
	**MCV** (42.20 – 59.20 fL)	48.00 ± 0.00	-	-	46.95 ± 1.34	-	-
	**MPV**	6.50 ± 0.57	-	-	10.30 ± 5.09	-	-
	**CH**	14.00 ± 0.28	-	-	13.65 ± 0.07	-	-
	**CHCM**	29.20 ± 0.57	-	-	29.15 ± 1.06	-	-
	**HDW**	1.72 ± 0.04	-	-	1.62 ± 0.10	-	-
	**PLT** (766.0 – 1657.0 K/uL)	856.60 ± 225.57	-	-	625.50 ± 770.04	-	-
	**LYMPH COUNT** (3.80 – 8.90 K/uL)	4.21 ± 0.45	-	-	3.48 ± 0.31	-	-
	**LYMPH %**	74.05 ± 16.05	-	-	80.55 ± 0.64	-	-
	**NEUT COUNT** (0.50 – 2.00 K/uL)	0.76 ± 0.28	-	-	0.56 ± 0.08	-	-
	**NEUT %**	13.00 ± 3.39	-	-	12.85 ± 0.64	-	-
	**MONO COUNT** (0.00 – 0.30 K/uL)	0.05 ± 0.01	-	-	0.05 ± 0.02	-	-
	**MONO %**	0.85 ± 0.07	-	-	1.05 ± 0.35	-	-
	**EOS COUNT** (0.00 – 0.40 K/uL)	0.09 ± 0.02	-	-	0.11 ± 0.04	-	-
	**EOS %**	1.50 ± 0.28	-	-	2.50 ± 0.99	-	-

	**BASO COUNT** (0.00 – 0.10 K/uL)	0.03 ± 0.01	-	-	0.02 ± 0.01	-	-
	**BASO %**	0.50 ± 0.14	-	-	0.40 ± 0.14	-	-
	**LUC COUNT**	0.62 ± 0.72	-	-	0.12 ± 0.04	-	-
	**LUC %**	10.10 ± 12.02	-	-	2.70 ± 0.85	-	-
	**RDW %** (11.70 – 15.10)	13.65 ± 1.63	-	-	12.10 ± 0.14	-	-
	**HCT %** (36.70 – 46.80)	43.15 ± 1.34	-	-	46.50 ± 0.71	-	-
	**RETICS %**	3.90 ± 0.52	-	-	3.30 ± 0.37	-	-
**Day 1**	**WBC** (3.20 – 12.70 K/uL)	4.72 ± 0.15	7.72 ± 4.99	2.79 ± 3.21	3.61 ± 2.49	4.67 ± 0.10	4.26 ± 0.00
	**RBC** (7.00 – 10.10 M/uL)	8.68 ± 0.15	9.27 ± 0.86	4.65 ± 6.54	6.21 ± 4.68	8.65 ± 0.28	8.51 ± 0.00
	**HGB** (11.80 – 14.90 g/dL)	13.13 ± 0.21	14.03 ± 1.19	8.35 ± 8.98	9.53 ± 7.39	13.60 ± 0.42	13.30 ± 0.00
	**MCH** (13.80 – 18.40 pg)	15.13 ± 0.15	15.17 ± 0.21	2.20 ± 0.00	14.67 ± 1.45	15.75 ± 0.07	15.60 ± 00
	**MCHC** (31.00 – 34.70 g/dL)	31.30 ± 0.36	31.47 ± 0.78	4.40 ± 0.00	29.50 ± 3.24	32.50 ± 0.28	32.50 ± 0.00
	**MCV** (42.20 – 59.20 fL)	48.37 ± 0.31	48.17 ± 0.81	47.50 ± 1.70	49.77 ± 0.60	48.50 ± 0.28	48.00 ± 0.00
	**MPV**	7.37 ± 0.40	10.07 ± 3.58	11.65 ± 5.02	8.73 ± 1.71	11.20 ± 4.53	11.80 ± 0.00
	**CH**	13.90 ± 0.44	13.67 ± 0.06	13.65 ± 0.07	14.37 ± 0.25	14.15 ± 0.07	14.10 ± 0.00
	**CHCM**	31.30 ± 0.36	31.47 ± 0.78	-	29.50 ± 3.24	32.50 ± 0.28	32.50 ± 0.00
	**HDW**	1.68 ± 0.08	1.60 ± 0.08	2.86 ± 1.85	1.64 ± 0.08	1.67 ± 0.01	1.71 ± 0.00
	**PLT** (766.0 – 1657.0 K/uL)	1382.00 ± 289.34	823.00 ± 544.51	400.50 ± 560.74	530.67 ± 435.31	527.50 ± 420.73	451.00 ± 0.00
	**LYMPH COUNT** (3.80 – 8.90 K/uL)	3.70 ± 0.42	6.85 ± 4.49	2.37 ± 2.64	3.02 ± 2.11	3.95 ± 0.30	2.61 ± 0.00
	**LYMPH %**	78.67 ± 9.65	88.30 ± 0.92	89.80 ± 8.49	84.33 ± 3.95	84.55 ± 4.74	61.10 ± 0.00
	**NEUT COUNT** (0.50 – 2.00 K/uL)	0.76 ± 0.39	0.58 ± 0.25	0.21 ± 0.28	0.34 ± 0.23	0.45 ± 0.13	0.83 ± 0.00
	**NEUT %**	16.13 ± 8.10	8.40 ± 2.17	4.65 ± 4.45	9.33 ± 1.43	9.55 ± 3.04	19.50 ± 0.00

	**MONO COUNT** (0.00 – 0.30 K/uL)	0.037 ± 0.006	0.037 ± 0.040	0.025 ± 0.035	0.020 ± 0.020	0.025 ± 0.007	0.040 ± 0.00
	**MONO %**	0.73 ± 0.21	0.40 ± 0.20	0.45 ± 0.64	0.63 ± 0.25	0.55 ± 0.07	0.90 ± 0.00
	**EOS COUNT** (0.00 – 0.40 K/uL)	0.10 ± 0.01	0.11 ± 0.09	0.08 ± 0.10	0.10 ± 0.07	0.09 ± 0.03	0.60 ± 0.00
	**EOS %**	2.23 ± 0.21	1.30 ± 0.30	2.70 ± 0.42	2.63 ± 0.60	1.90 ± 0.57	4.20 ± 0.00
	**BASO COUNT** (0.00 – 0.10 K/uL)	0.003 ± 0.006	0.013 ± 0.015	0.005 ± 0.007	0.007 ± 0.006	0.010 ± 0.00	0.110 ± 0.00
	**BASO %**	0.07 ± 0.06	0.20 ± 0.10	0.20 ± 0.00	0.17 ± 0.15	0.25 ± 0.07	2.70 ± 0.00
	**LUC COUNT**	0.10 ± 0.08	0.13 ± 0.12	0.11 ± 0.15	0.12 ± 0.10	0.15 ± 0.06	0.07 ± 0.00
	**LUC %**	2.20 ± 1.56	1.47 ± 0.84	2.10 ± 2.97	2.93 ± 1.79	3.20 ± 1.27	1.70 ± 0.00
	**RDW %** (11.70 – 15.10)	13.47 ± 1.02	12.27 ± 0.15	13.95 ± 2.90	12.87 ± 0.40	12.35 ± 0.07	13.30 ± 0.00
	**HCT %** (36.70 – 46.80)	41.97 ± 0.47	44.63 ± 4.36	22.60 ± 31.82	30.70 ± 23.13	41.95 ± 1.63	40.90 ± 0.00
	**RETICS %**	3.33 ± 0.20	3.76 ± 0.29	3.53 ± 0.20	4.41 ± 0.85	3.38 ± 0.09	4.14 ± 0.00
**Day 4**	**WBC** (3.20 – 12.70 K/uL)	5.21 ± 6.13	3.36 ± 4.31	-	6.43 ± 2.84	2.17 ± 2.55	-
	**RBC** (7.00 – 10.10 M/uL)	5.49 ± 6.10	4.80 ± 5.6	-	7.84 ± 0.02	0.89 ± 0.11	-
	**HGB** (11.80 – 14.90 g/dL)	8.60 ± 8.91	7.30 ± 8.34	-	12.75 ± 0.21	2.30 ± 1.27	-
	**MCH** (13.80 – 18.40 pg)	17.45 ± 3.18	16.60 ± 2.26	-	16.25 ± 0.21	27.10 ± 17.25	-
	**MCHC** (31.00 – 34.70 g/dL)	37.37 ± 8.56	35.75 ± 5.44	-	33.20 ± 1.84	56.75 ± 35.14	-
	**MCV** (42.20 – 59.20 fL)	46.40 ± 2.26	46.50 ± 0.85	-	49.00 ± 2.12	47.35 ± 1.20	-
	**MPV**	13.85 ± 7.99	27.60 ± 12.59	-	10.30 ± 2.97	25.80 ± 0.85	-
	**CH**	13.40 ± 0.14	13.45 ± 0.07	-	14.35 ± 0.35	14.00 ± 0.14	-
	**CHCM**	28.90 ± 1.13	29.05 ± 0.35	-	29.30 ± 0.42	29.65 ± 1.06	-
	**HDW**	1.89 ± 0.45	2.12 ± 0.61	-	1.67 ± 0.06	1.99 ± 0.26	-
	**PLT** (766.0 – 1657.0 K/uL)	563.30 ± 733.27	120.50 ± 150.61	-	425.50 ± 137.89	24.00 ± 9.90	-

	**LYMPH COUNT** (3.80 – 8.90 K/uL)	4.40 ± 5.07	2.90 ± 3.68	-	5.25 ± 2.16	1.94 ± 2.31	-
	**LYMPH %**	88.40 ± 6.79	90.75 ± 6.86	-	82.25 ± 2.76	88.70 ± 1.70	-
	**NEUT COUNT** (0.50 – 2.00 K/uL)	0.28 ± 0.37	0.17 ± 0.24	-	0.55 ± 0.32	0.06 ± 0.05	-
	**NEUT %**	3.95 ± 2.33	3.10 ± 3.11	-	8.15 ± 1.34	4.50 ± 3.39	-
	**MONO COUNT** (0.00 – 0.30 K/uL)	0.07 ± 0.08	0.04 ± 0.05	-	0.07 ± 0.04	0.01 ± 0.01	-
	**MONO %**	1.40 ± 0.14	1.00 ± 0.14	-	0.95 ± 0.07	0.50 ± 0.14	-
	**EOS COUNT** (0.00 – 0.40 K/uL)	0.12 ± 0.13	0.10 ± 0.13	-	0.25 ± 0.11	0.03 ± 0.03	-
	**EOS %**	1.95 ± 0.35	2.40 ± 0.85	-	3.80 ± 0.00	1.75 ± 0.64	-
	**BASO COUNT** (0.00 – 0.10 K/uL)	0.01 ± 0.01	0.04 ± 0.05	-	0.02 ± 0.01	0.04 ± 0.06	-
	**BASO %**	0.10 ± 0.14	0.75 ± 0.49	-	0.25 ± 0.07	1.15 ± 1.20	-
	**LUC COUNT**	0.34 ± 0.47	0.12 ± 0.16	-	0.32 ± 0.21	0.09 ± 0.11	-
	**LUC %**	4.10 ± 4.10	2.00 ± 2.26	-	4.65 ± 1.34	3.40 ± 1.27	-
	**RDW %** (11.70 – 15.10)	12.45 ± 0.78	12.50 ± 0.85	-	12.85 ± 0.64	13.15 ± 0.07	-
	**HCT %** (36.70 – 46.80)	26.20 ± 29.56	22.50 ± 26.73	-	38.40 ± 1.56	4.20 ± 0.42	-
	**RETICS %**	3.70 ± 0.34	3.21 ± 0.10	-	3.91 ± 0.60	3.72 ± 0.04	-
**Day 7**	**WBC** (3.20 – 12.70 K/uL)	8.83 ± 3.83	8.65 ± 2.04	-	3.39 ± 0.66	5.07 ± 2.44	-
	**RBC** (7.00 – 10.10 M/uL)	9.42 ± 0.68	9.48 ± 0.30	-	8.69 ± 0.33	8.85 ± 0.08	-
	**HGB** (11.80 – 14.90 g/dL)	14.33 ± 0.87	14.03 ± 0.32	-	13.17 ± 0.06	13.57 ± 0.12	-
	**MCH** (13.80 – 18.40 pg)	15.23 ± 0.23	14.83 ± 0.15	-	15.20 ± 0.53	15.30 ± 0.10	-
	**MCHC** (31.00 – 34.70 g/dL)	31.93 ± 0.65	30.83 ± 0.25	-	31.07 ± 1.42	31.67 ± 0.32	-
	**MCV** (42.20 – 59.20 fL)	47.67 ± 0.38	48.07 ± 0.55	-	48.87 ± 1.05	48.33 ± 0.55	-
	**MPV**	7.57 ± 0.25	7.00 ± 0.20	-	7.87 ± 0.81	7.27 ± 0.40	-

	**CH**	13.80 ± 0.10	13.83 ± 0.06	-	14.07 ± 0.21	14.13 ± 0.15	-
	**CHCM**	29.00 ± 0.10	28.83 ± 0.23	-	28.87 ± 0.91	29.27 ± 0.35	-
	**HDW**	1.61 ± 0.02	1.63 ± 0.03	-	1.65 ± 0.01	1.61 ± 0.07	-
	**PLT** (766.0 – 1657.0 K/uL)	820.33 ± 159.8	691.67 ± 226.09	-	364.33 ± 183.92	618.33 ± 270.48	-
	**LYMPH COUNT** (3.80 – 8.90 K/uL)	7.47 ± 3.20	7.60 ± 1.90	-	2.78 ± 0.55	4.23 ± 2.00	-
	**LYMPH %**	84.77 ± 0.57	87.73 ± 1.96	-	82.17 ± 5.52	83.50 ± 0.62	-
	**NEUT COUNT** (0.50 – 2.00 K/uL)	0.69 ± 0.27	0.54 ± 0.16	-	0.31 ± 0.04	0.45 ± 0.22	-
	**NEUT %**	7.90 ± 0.61	6.37 ± 1.32	-	9.27 ± 0.81	8.97 ± 0.50	-
	**MONO COUNT** (0.00 – 0.30 K/uL)	0.09 ± 0.04	0.10 ± 0.03	-	0.02 ± 0.01	0.03 ± 0.01	-
	**MONO %**	1.07 ± 0.21	1.10 ± 0.00	-	0.67 ± 0.06	0.67 ± 0.21	-
	**EOS COUNT** (0.00 – 0.40 K/uL)	0.31 ± 0.13	0.22 ± 0.03	-	0.21 ± 0.23	0.15 ± 0.08	-
	**EOS %**	3.50 ± 0.46	2.63 ± 0.76	-	6.07 ± 6.18	3.00 ± 0.36	-
	**BASO COUNT** (0.00 – 0.10 K/uL)	0.023 ± 0.02	0.02 ± 0.01	-	0.017 ± 0.01	0.017 ± 0.02	-
	**BASO %**	0.23 ± 0.12	0.20 ± 0.10	-	0.50 ± 0.10	0.27 ± 0.21	-
	**LUC COUNT**	0.24 ± 0.18	0.17 ± 0.02	-	0.05 ± 0.04	0.19 ± 0.12	-
	**LUC %**	2.57 ± 0.72	2.03 ± 0.31	-	1.37 ± 0.76	3.63 ± 0.51	-
	**RDW %** (11.70 – 15.10)	12.00 ± 0.10	12.50 ± 0.87	-	12.73 ± 0.12	12.50 ± 0.44	-
	**HCT %** (36.70 – 46.80)	44.90 ± 3.20	45.57 ± 1.42	-	42.47 ± 1.95	42.73 ± 0.75	-
	**RETICS %**	3.37 ± 0.08	3.88 ± 0.75	-	3.72 ± 0.55	3.37 ± 0.25	-
	**WBC** (3.20 – 12.70 K/uL)	8.43 ± 2.90	7.90 ± 2.41	6.83 ± 1.89	2.24 ± 1.28	3.88 ± 1.42	4.83 ± 1.46
	**RBC** (7.00 – 10.10 M/uL)	7.92 ± 2.49	9.14 ± 0.33	9.38 ± 0.16	7.11 ± 0.50	8.92 ± 0.26	7.81 ± 1.33

**Day 14**	**HGB** (11.80 – 14.90 g/dL)	9.43 ± 7.08	13.23 ± 1.25	14.13 ± 0.68	11.07 ± 0.71	14.13 ± 0.25	12.37 ± 2.05
	**MCH** (13.80 – 18.40 pg)	10.47 ± 6.90	14.43 ± 0.91	15.07 ± 0.55	15.60 ± 0.26	15.83 ± 0.23	15.83 ± 0.15
	**MCHC** (31.00 – 34.70 g/dL)	20.80 ± 13.95	28.50 ± 1.99	29.77 ± 1.24	30.10 ± 0.26	30.30 ± 0.78	29.73 ± 0.70
	**MCV** (42.20 – 59.20 fL)	51.50 ± 2.50	50.70 ± 0.78	50.57 ± 0.25	51.73 ± 0.45	52.33 ± 0.55	53.37 ± 0.67
	**MPV**	7.43 ± 0.29	7.33 ± 0.32	7.37 ± 0.55	13.73 ± 4.46	7.53 ± 0.49	8.17 ± 0.35
	**CH**	13.53 ± 0.21	13.57 ± 0.15	13.70 ± 0.10	14.17 ± 0.15	14.20 ± 0.17	14.20 ± 0.20
	**CHCM**	26.37 ± 0.84	26.87 ± 0.15	27.17 ± 0.21	27.43 ± 0.06	27.17 ± 0.50	26.67 ± 0.67
	**HDW**	1.78 ± 0.34	1.60 ± 0.05	1.56 ± 0.07	1.55 ± 0.03	1.55 ± 0.09	1.56 ± 0.03
	**PLT** (766.0 – 1657.0 K/uL)	1008.00 ± 348.18	998.33 ± 154.86	1177.00 ± 663.45	97.33 ± 63.80	726.33 ± 314.31	458.00 ± 230.75
	**LYMPH COUNT** (3.80 – 8.90 K/uL)	5.21 ± 1.28	6.16 ± 2.26	5.71 ± 2.00	1.99 ± 1.13	3.37 ± 1.21	3.71 ± 1.88
	**LYMPH %**	64.80 ± 15.11	77.60 ± 10.05	82.73 ± 7.46	89.00 ± 0.62	86.90 ± 1.49	72.87 ± 20.76
	**NEUT COUNT** (0.50 – 2.00 K/uL)	2.38 ± 1.92	1.14 ± 0.94	0.43 ± 0.05	0.13 ± 0.10	0.25 ± 0.08	0.38 ± 0.09
	**NEUT %**	25.10 ± 16.54	14.87 ± 10.20	6.77 ± 2.23	5.50 ± 1.14	6.57 ± 0.31	8.50 ± 3.65
	**MONO COUNT** (0.00 – 0.30 K/uL)	0.09 ± 0.04	0.09 ± 0.03	0.05 ± 0.02	0.01 ± 0.01	0.03 ± 0.02	0.04 ± 0.02
	**MONO %**	1.00 ± 0.10	1.17 ± 0.40	0.67 ± 0.12	0.40 ± 0.20	0.77 ± 0.31	0.80 ± 0.20
	**EOS COUNT** (0.00 – 0.40 K/uL)	0.51 ± 0.33	0.31 ± 0.12	0.40 ± 0.42	0.04 ± 0.01	0.11 ± 0.04	0.45 ± 0.61
	**EOS %**	6.50 ± 3.64	3.80 ± 0.46	6.70 ± 7.41	1.77 ± 0.72	2.90 ± 0.35	13.20 ± 19.84
	**BASO COUNT** (0.00 – 0.10 K/uL)	0.01 ± 0.00	0.01 ± 0.01	0.01 ± 0.00	0.01 ± 0.01	0.01 ± 0.01	0.05 ± 0.06
	**BASO %**	0.10 ± 0.00	0.13 ± 0.06	0.13 ± 0.06	0.23 ± 0.12	0.13 ± 0.06	0.93 ± 0.92
	**LUC COUNT**	0.22 ± 0.23	0.19 ± 0.08	0.23 ± 0.19	0.07 ± 0.04	0.12 ± 0.08	0.20 ± 0.18
	**LUC %**	2.43 ± 1.99	2.47 ± 0.68	3.00 ± 1.73	3.23 ± 1.53	2.70 ± 1.28	3.73 ± 2.70
	**RDW %** (11.70 – 15.10)	15.17 ± 3.38	13.48 ± 0.81	13.00 ± 0.69	12.40 ± 0.70	12.60 ± 0.26	12.70 ± 0.26

	**HCT %** (36.70 – 46.80)	40.43 ± 11.15	46.33 ± 1.36	47.43 ± 0.57	36.77 ± 2.63	46.73 ± 1.82	41.70 ± 7.27
	**RETICS %**	4.45 ± 1.66	3.59 ± 0.12	3.36 ± 0.12	3.22 ± 1.26	4.16 ± 0.59	3.97 ± 0.38
**Day 30**	**WBC** (3.20 – 12.70 K/uL)	7.99 ± 2.15	6.91 ± 0.54	3.54 ± 3.08	3.72 ± 3.28	3.21 ± 4.56	5.24 ± 3.62
	**RBC** (7.00 – 10.10 M/uL)	9.37 ± 0.32	8.51 ± 0.14	5.83 ± 5.05	6.09 ± 4.84	3.19 ± 4.70	8.17 ± 1.22
	**HGB** (11.80 – 14.90 g/dL)	13.93 ± 0.25	13.20 ± 0.56	8.93 ± 7.74	9.27 ± 7.42	6.10 ± 7.08	12.93 ± 2.08
	**MCH** (13.80 – 18.40 pg)	14.90 ± 0.26	15.53 ± 0.64	10.23 ± 8.86	14.93 ± 0.64	45.30 ± 49.97	15.80 ± 0.62
	**MCHC** (31.00 – 34.70 g/dL)	31.87 ± 0.76	32.43 ± 1.33	21.37 ± 18.52	32.00 ± 0.10	96.17 ± 108.20	32.83 ± 1.33
	**MCV** (42.20 – 59.20 fL)	46.73 ± 0.45	47.83 ± 0.21	31.93 ± 27.66	46.67 ± 1.83	48.13 ± 2.24	48.20 ± 0.17
	**MPV**	7.50 ± 0.10	8.00 ± 0.62	4.77 ± 4.14	13.00 ± 7.97	15.17 ± 4.50	11.13 ± 5.54
	**CH**	13.50 ± 0.20	13.67 ± 0.15	9.23 ± 8.00	13.93 ± 0.06	14.27 ± 0.31	14.07 ± 0.12
	**CHCM**	28.87 ± 0.32	28.67 ± 0.40	19.23 ± 16.66	29.90 ± 1.18	29.63 ± 0.72	29.23 ± 0.35
	**HDW**	1.64 ± 0.05	1.67 ± 0.04	1.07 ± 0.92	1.75 ± 0.22	1.84 ± 0.17	1.63 ± 0.04
	**PLT** (766.0 – 1657.0 K/uL)	1132.67 ± 117.78	1000.33 ± 202.40	770.67 ± 669.85	336.00 ± 277.35	122.00 ± 180.14	619.33 ± 510.89
	**LYMPH COUNT** (3.80 – 8.90 K/uL)	6.84 ± 1.92	5.85 ± 0.49	3.01 ± 2.62	3.15 ± 2.80	2.43 ± 3.32	4.56 ± 3.13
	**LYMPH %**	85.40 ± 1.56	84.70 ± 2.12	56.70 ± 49.12	85.00 ± 0.70	85.50 ± 10.46	87.97 ± 2.43
	**NEUT COUNT** (0.50 – 2.00 K/uL)	0.46 ± 0.13	0.48 ± 0.10	0.30 ± 0.27	0.31 ± 0.28	0.20 ± 0.31	0.30 ± 0.23
	**NEUT %**	5.73 ± 0.75	7.03 ± 1.53	5.63 ± 5.09	7.17 ± 2.06	4.60 ± 3.64	5.17 ± 0.91
	**MONO COUNT** (0.00 – 0.30 K/uL)	0.11 ± 0.04	0.08 ± 0.01	0.04 ± 0.04	0.05 ± 0.05	0.03 ± 0.04	0.04 ± 0.03
	**MONO %**	1.33 ± 0.35	1.20 ± 0.20	0.83 ± 0.72	2.43 ± 2.06	0.87 ± 0.15	0.87 ± 0.06
	**EOS COUNT** (0.00 – 0.40 K/uL)	0.31 ± 0.08	0.29 ± 0.05	0.11 ± 0.10	0.13 ± 0.11	0.10 ± 0.14	0.18 ± 0.18
	**EOS %**	3.97 ± 1.00	4.17 ± 0.47	2.13 ± 1.87	3.97 ± 0.76	2.60 ± 0.50	3.20 ± 2.70

	**BASO COUNT** (0.00 – 0.10 K/uL)	0.02 ± 0.01	0.02 ± 0.01	0.01 ± 0.01	0.01 ± 0.01	0.01 ± 0.02	0.02 ± 0.02
	**BASO %**	0.27 ± 0.12	0.17 ± 0.12	0.10 ± 0.10	0.13 ± 0.12	0.37 ± 0.21	0.33 ± 0.15
	**LUC COUNT**	0.25 ± 0.06	0.19 ± 0.05	0.07 ± 0.06	0.06 ± 0.06	0.43 ± 0.73	0.13 ± 0.14
	**LUC %**	3.33 ± 1.08	2.73 ± 0.76	1.23 ± 1.07	1.23 ± 1.25	6.07 ± 7.83	2.43 ± 1.10
	**RDW %** (11.70 – 15.10)	12.43 ± 0.31	13.30 ± 0.75	8.20 ± 7.10	12.67 ± 0.60	13.13 ± 0.12	12.17 ± 0.12
	**HCT %** (36.70 – 46.80)	43.77 ± 1.70	40.70 ± 0.56	27.93 ± 24.20	28.93 ± 23.15	16.03 ± 23.98	39.37 ± 5.71
	**RETICS %**	3.63 ± 0.35	3.99 ± 0.37	2.30 ± 2.00	3.15 ± 1.14	3.89 ± 1.42	3.40 ± 0.39
**Day 90**	**WBC** (3.20 – 12.70 K/uL)	1.24 ± 0.00	5.61 ± 0.00	5.79 ± 0.00	0.48 ± 0.00	1.76 ± 1.95	0.67 ± 0.00
	**RBC** (7.00 – 10.10 M/uL)	1.00 ± 0.00	7.43 ± 0.00	8.02 ± 0.00	0.50 ± 0.00	4.24 ± 4.66	0.82 ± 0.00
	**HGB** (11.80 – 14.90 g/dL)	1.40 ± 0.00	12.20 ± 0.00	12.50 ± 0.00	0.80 ± 0.00	6.65 ± 7.85	1.30 ± 0.00
	**MCH** (13.80 – 18.40 pg)	14.00 ± 0.00	16.50 ± 0.00	15.60 ± 0.00	16.10 ± 0.00	14.20 ± 2.83	16.40 ± 0.00
	**MCHC** (31.00 – 34.70 g/dL)	30.50 ± 0.00	34.50 ± 0.00	33.20 ± 0.00	35.00 ± 0.00	30.75 ± 6.15	35.50 ± 0.00
	**MCV** (42.20 – 59.20 fL)	64.10 ± 0.00	47.70 ± 0.00	47.00 ± 0.00	46.10 ± 0.00	46.10 ± 0.00	46.30 ± 0.00
	**MPV**	27.20 ± 0.00	21.90 ± 0.00	21.70 ± 0.00	29.20 ± 0.00	28.55 ± 2.47	30.30 ± 0.00
	**CH**	13.80 ± 0.00	14.20 ± 0.00	13.60 ± 0.00	13.70 ± 0.00	14.00 ± 0.14	14.00 ± 0.00
	**CHCM**	30.00 ± 0.00	29.80 ± 0.00	28.90 ± 0.00	29.80 ± 0.00	30.35 ± 0.21	30.30 ± 0.00
	**HDW**	1.81 ± 0.00	1.73 ± 0.00	1.61 ± 0.00	1.75 ± 0.00	1.90 ± 0.11	2.20 ± 0.00
	**PLT** (766.0 – 1657.0 K/uL)	7.00 ± 0.00	94.00 ± 0.00	79.00 ± 0.00	5.00 ± 0.00	35.00 ± 32.53	26.00 ± 0.00
	**LYMPH COUNT** (3.80 – 8.90 K/uL)	1.15 ± 0.00	5.07 ± 0.00	5.26 ± 0.00	0.43 ± 0.00	1.60 ± 1.75	0.59 ± 0.00
	**LYMPH %**	92.90 ± 0.00	90.40 ± 0.00	90.80 ± 0.00	89.90 ± 0.00	92.05 ± 2.47	88.90 ± 0.00
	**NEUT COUNT** (0.50 – 2.00 K/uL)	0.03 ± 0.00	0.22 ± 0.00	0.27 ± 0.00	0.01 ± 0.00	0.07 ± 0.08	0.03 ± 0.00
	**NEUT %**	2.40 ± 0.00	4.00 ± 0.00	4.60 ± 0.00	3.00 ± 0.00	2.55 ± 1.63	4.80 ± 0.00

	**MONO COUNT** (0.00 – 0.30 K/uL)	0.01 ± 0.00	0.03 ± 0.00	0.05 ± 0.00	0.00 ± 0.00	0.01 ± 0.00	0.01 ± 0.00
	**MONO %**	0.40 ± 0.00	0.60 ± 0.00	0.90 ± 0.00	0.70 ± 0.00	0.90 ± 0.71	1.40 ± 0.00
	**EOS COUNT** (0.00 – 0.40 K/uL)	0.01 ± 0.00	0.08 ± 0.00	0.09 ± 0.00	0.01 ± 0.00	0.02 ± 0.01	0.01 ± 0.00
	**EOS %**	0.60 ± 0.00	1.30 ± 0.00	1.50 ± 0.00	1.40 ± 0.00	1.55 ± 0.78	2.10 ± 0.00
	**BASO COUNT** (0.00 – 0.10 K/uL)	0.00 ± 0.00	0.06 ± 0.00	0.03 ± 0.00	0.00 ± 0.00	0.01 ± 0.01	0.00 ± 0.00
	**BASO %**	0.20 ± 0.00	1.00 ± 0.00	0.40 ± 0.00	0.00 ± 0.00	0.40 ± 0.57	0.20 ± 0.00
	**LUC COUNT**	0.04 ± 0.00	0.15 ± 0.00	0.10 ± 0.00	0.02 ± 0.00	0.07 ± 0.08	0.02 ± 0.00
	**LUC %**	3.50 ± 0.00	2.70 ± 0.00	1.70 ± 0.00	5.10 ± 0.00	2.60 ± 1.70	2.60 ± 0.00
	**RDW %** (11.70 – 15.10)	13.10 ± 0.00	13.30 ± 0.00	12.40 ± 0.00	12.30 ± 0.00	13.10 ± 0.28	13.30 ± 0.00
	**HCT %** (36.70 – 46.80)	4.60 ± 0.00	35.50 ± 0.00	37.70 ± 0.00	2.30 ± 0.00	19.50 ± 21.50	3.80 ± 0.00
	**RETICS %**	3.45 ± 0.00	4.00 ± 0.00	3.27 ± 0.00	3.23 ± 0.00	2.88 ± 0.28	4.54 ± 0.00

Table S3. Hematology analysis from male and female mice at different days of treatment (data plotted as mean, n = 3). Complete blood cell count parameters displayed with reference range to facilitate the assessment of different treatment groups compared to the untreated healthy mice.

Table S4. Histological findings on selected organs from non-tumor bearing mice (n = 1 - 6) ten weeks post-treatment.

**Cohorts**	**Organs**	**Organ Histology**	
		**Normal**	**With lesions (type & degree)**
	Heart	6 out of 6	None
	Kidney	6 out of 6	None
	Lung	6 out of 6	None
	Liver	5 out of 6	1 out of 6 (hepatocellular lipodosis, midzonal: 2MF)
Control	Urinary bladder	6 out of 6	None
	Adrenal gland	5 out of 5	None
	Mesenteric lymph node	3 out of 3	None
	Pancreas	4 out of 4	None
	Heart	5 out of 5	None
	Kidney	3 out of 5	2 out of 5 (Nephroblastoma, hydronephrosis: 3U; perivascular lymphocytic infiltrates: 1MF)
	Lung	5 out of 5	None
	Liver	3 out of 5	2 out of 5 (Perivascular lymphocytic infiltrates: 3D; microabscesses: 3MF)
Anti-CD40	Urinary bladder	5 out of 5	None
	Adrenal gland	4 out of 5	1 out of 5 (Adrenal cortex, zona fasciculata lipidosis: 2D)
	Mesenteric lymph node	4 out of 4	None
	Pancreas	1 out of 1	None
	Heart	6 out of 6	None

iSRB	Kidney	6 out of 6	None
	Lung	6 out of 6	None
	Liver	4 out of 6	2 out of 6 (Perivascular lymphocytic infiltrates: 2D; microabscesses: 1MF)
	Urinary bladder	6 out of 6	None
	Adrenal gland	5 out of 5	None
	Mesenteric lymph node	3 out of 3	None
	Pancreas	3 out of 3	None
iSRB-Anti-CD40	Heart	6 out of 6	None
	Kidney	5 out of 6	1 out of 6 (Renal cortex infarction: 2F)
	Lung	5 out of 6	1 out of 6 (Perivascular lymphocytic aggregates: 2MF)
	Liver	6 out of 6	None
	Urinary bladder	6 out of 6	None
	Adrenal gland	6 out of 6	None
	Mesenteric lymph node	3 out of 3	None
	Pancreas	1 out of 1	None

Table S4. Histopathology reports ten weeks post-treatment for the collected organs such as heart, lung, liver, Urinary bladder, adrenal gland, mesenteric lymph node, pancreas, spleen, and two kidneys. Organs were collected right after blood collection from cardiac puncture, needle passing from the abdomen puncturing through the liver. Lesions can be any damages incurred by the organ, such as Hemorrhage intra-alveolar (likely iatrogenic, due to the method of euthanasia) or Hepatocellular swelling, with intracytoplasmic microvesicular lipidosis. Most mice tissues incurred no lesions except the liver across all cohorts. The common lesions found in the liver include 1) Micro granulomas, characterized by small nodular accumulations (up to 30 cells) of lymphocytes, histiocytes, a few neutrophils, and entrapped degenerating hepatocytes; 2) Microabscesses, characterized by small nodular accumulations of neutrophils, lymphocytes, and histiocytes (up to 30 cells) with entrapped degenerating hepatocytes; 3) Extramedullary hematopoiesis - clonal colonies of myeloid precursors; 4) Hepatocytes, individual cell necrosis (dropout). Scoring Definitions: (0 = No finding; 1 = Minimal; 2 = Mild; 3 = Moderate; 4 = Marked; 5 = Severe; N = Normal; M = Missing; B = Bilateral; MF = Multifocal; F = Focal; D = Diffuse; U = Unilateral; B = Bilateral)

Table S5. Histological findings on selected organs from non-tumor bearing mice at different time points post-treatment (n = 3/group/time point).

Time points	Cohorts	Female Mice					Lesions Found	
		Heart	Lung	Spleen	Kidneys	Liver	Ratio of tissues showing No finding	Lesions scores
Day 1	No Treatment	3 out of 3	2 out of 3	3 out of 3	3 out of 3	2 out of 3	13 OUT OF 15	1MF / 2MF
	iSRB	3 out of 3	1 out of 3	3 out of 3	3 out of 3	2 out of 3	12 OUT OF 15	1MF / 2MF
	iSRB_Ab-CD40	3 out of 3	3 out of 3	3 out of 3	3 out of 3	1 out of 3	13 OUT OF 15	1MF
Day 4	iSRB	2 out of 2	1 out of 2	2 out of 2	2 out of 2	1 out of 2	8 OUT OF 10	2MF / 3MF
	iSRB_Ab-CD40	2 out of 2	1 out of 2	2 out of 2	2 out of 2	1 out of 2	8 OUT OF 10	2F / 2MF
Day 7	iSRB	3 out of 3	3 out of 3	3 out of 3	3 out of 3	3 out of 3	15 OUT OF 15	N
	iSRB_Ab-CD40	3 out of 3	2 out of 3	3 out of 3	3 out of 3	0 out of 3	11 OUT OF 15	1MF / 2MF
Day 14	No Treatment	3 out of 3	2 out of 3	2 out of 3	3 out of 3	0 out of 3	10 OUT OF 15	1MF / 2D
	iSRB	3 out of 3	2 out of 3	3 out of 3	3 out of 3	1 out of 3	12 OUT OF 15	1F / 1MF
	iSRB_Ab-CD40	3 out of 3	1 out of 2	3 out of 3	3 out of 3	0 out of 3	10 OUT OF 14	M / 1MF / 2MF
Day 30	No Treatment	3 out of 3	1 out of 3	3 out of 3	3 out of 3	1 out of 3	11 OUT OF 15	1MF / 2MF
	iSRB	3 out of 3	1 out of 3	3 out of 3	3 out of 3	2 out of 3	12 OUT OF 15	1MF / 2MF
	iSRB_Ab-CD40	3 out of 3	3 out of 3	3 out of 3	3 out of 3	0 out of 3	12 OUT OF 15	2MF
Day 90	No Treatment	3 out of 3	2 out of 3	3 out of 3	0 out of 3	2 out of 3	10 OUT OF 15	1MF
	iSRB	3 out of 3	2 out of 3	3 out of 3	3 out of 3	1 out of 3	12 OUT OF 15	3D / 1MF
	iSRB_Ab-CD40	3 out of 3	2 out of 3	3 out of 3	3 out of 3	0 out of 3	11 OUT OF 15	1MF

Time points	Cohorts	Male Mice					Lesions Found	
		Heart	Lung	Spleen	Kidneys	Liver	Ratio of tissues showing No finding	Lesions scores
Day 1	No Treatment	3 out of 3	2 out of 3	3 out of 3	3 out of 3	0 out of 3	11 OUT OF 15	1MF / 2MF
	iSRB	3 out of 3	2 out of 3	3 out of 3	3 out of 3	3 out of 3	14 OUT OF 15	2MF
	iSRB_Ab-CD40	3 out of 3	3 out of 3	3 out of 3	3 out of 3	2 out of 3	14 OUT OF 15	1MF
Day 4	iSRB	2 out of 2	2 out of 2	2 out of 2	2 out of 2	1 out of 2	9 OUT OF 10	2F
	iSRB_Ab-CD40	2 out of 2	2 out of 2	2 out of 2	1 out of 2	1 out of 2	8 OUT OF 10	2MF / 3U
Day 7	iSRB	3 out of 3	3 out of 3	3 out of 3	3 out of 3	2 out of 3	14 OUT OF 15	2MF
	iSRB_Ab-CD40	3 out of 3	2 out of 3	3 out of 3	2 out of 3	2 out of 3	12 OUT OF 15	2MF / 3MF
Day 14	No Treatment	3 out of 3	3 out of 3	3 out of 3	3 out of 3	1 out of 3	13 OUT OF 15	1D / 1MF
	iSRB	3 out of 3	2 out of 3	3 out of 3	3 out of 3	3 out of 3	14 OUT OF 15	2F
	iSRB_Ab-CD40	3 out of 3	2 out of 3	3 out of 3	3 out of 3	1 out of 3	12 OUT OF 15	2F / 1MF / 2MF
Day 30	No Treatment	3 out of 3	3 out of 3	3 out of 3	3 out of 3	2 out of 3	14 OUT OF 15	2MF
	iSRB	3 out of 3	3 out of 3	3 out of 3	3 out of 3	1 out of 3	13 OUT OF 15	2MF
	iSRB_Ab-CD40	3 out of 3	2 out of 3	3 out of 3	3 out of 3	2 out of 3	13 OUT OF 15	2MF
Day 90	No Treatment	3 out of 3	3 out of 3	2 out of 3	2 out of 3	0 out of 3	10 OUT OF 15	2D / 1F / 1MF
	iSRB	3 out of 3	2 out of 3	3 out of 3	2 out of 3	1 out of 3	11 OUT OF 15	1F / 1MF / 2MF
	iSRB_Ab-CD40	3 out of 3	3 out of 3	3 out of 3	3 out of 3	1 out of 3	13 OUT OF 15	2D / 1F

Table S5. Histopathology report days 1, 4, 7, 14, 30, and 90 post-treatment for the collected organs such as heart, lung, liver, spleen, and two kidneys showing mild adverse effects. Common mild lesions found in these tissues include 1) Pulmonary Granulomas, characterized by inflammatory nodules replacing pre-existing parenchyma and containing a dense collection (up to 30 cells total) of macrophages, neutrophils and occasional multinucleate giant cells; 2) Periportal lymphocytic & histiocytic infiltrates; 3) Microabscesses, characterized by small nodular accumulations of neutrophils, lymphocytes, and histiocytes. Most mice tissues incurred no lesions except the liver across all cohorts. Scoring Definitions: (0 = No finding; 1 = Minimal; 2 = Mild; 3 = Moderate; 4 = Marked; 5 = Severe; N = Normal; M = Missing; B = Bilateral; MF = Multifocal; F = Focal; D = Diffuse; U = Unilateral; B = Bilateral)

Table S6. Histopathology report for Female Mice following vena cava blood sampling.

Mouse Accession	23019265	23019266	23019267	23019268	23019269	23019270
**Animal ID**	**No Treatment_#1 Day 7**	**No Treatment_#2 Day 7**	**No Treatment_#3 Day 7**	**No Treatment_#4 Day 7**	**No Treatment_#5 Day 7**	**iSRB_Anti-CD40_#1 Day 7**
**LIVER**	**N**	**N**				
**Microabscesses:** Mixed aggregates of inflammatory cells consisting primarily of neutrophils and fewer macrophages and/or lymphocytes, with one or more swollen and degenerating hepatocyes incorporated into the inflammatory focus.			**2MF**	**2MF**		
**Microgranulomas:** Mixed aggregates of inflammatory cells consisting up to 100 mixed lymphocytes, macrophages, random neutrophils, and a small number of incorporated hepatocytes that are often swollen and or degenerate.					**2MF**	
**Lymphocytic aggregates:** Aggregates of purely lymphocytes, up to 75 cells, located in perivascular and parenchymal locations throughout the liver.						**3MF**
**KIDNEY: RIGHT**	**N**	**N**	**N**	**N**	**N**	**N**
**KIDNEY: LEFT**	**N**	**N**	**N**	**N**	**N**	**N**

Mouse Accession	23019271	23019272	23019273	23019274
**Animal ID**	**iSRB_Anti-CD40_#2 Day 7**	**iSRB_Anti-CD40_#3 Day 7**	**iSRB_Anti-CD40_#4 Day 7**	**iSRB_Anti-CD40_#5 Day 7**
**LIVER**				
**Lymphocytic aggregates:** Liver portal and periportal connective tissue mildly expanded by infiltrates of low to Moderate number of lymphocytes. These cells often surround bile ducts. Additionally, small aggregates of pure lymphocytes are randomly distributed throughout the hepatic parenchyma.	**2MF**			
**Microgranulomas:** Mixed aggregates of inflammatory cells consisting up to 50 cells of lymphocytes, macrophages, random neutrophils, and a small number of incorporated hepatocytes randomly distributed throughout the hepatic parenchyma.		**2MF**		**2MF**
**Microgranulomas:** Mixed aggregates of inflammatory cells consisting up to 100 cells of lymphocytes, macrophages, a few admixed neutrophils, individual entrapped hepatocytes, and cellular debris.			**2MF**	
**KIDNEY: RIGHT**	**N**	**N**	**N**	**N**
**KIDNEY: LEFT**	**N**	**N**	**N**	**N**

Table S6. Pathology report corresponding to day seven post-treatment from harvested liver and kidney tissues from female mice (n = 5). Scoring Definitions: (0 = No finding; 1 = Minimal; 2 = Mild; 3 = Moderate; 4 = Marked; 5 = Severe; N = Normal; M = Missing; B = Bilateral; MF = Multifocal; F = Focal; D = Diffuse; U = Unilateral; B = Bilateral)
