# Supplementary material for: Pre-Clinical Investigations of the Pharmacodynamics of Immunogenic Smart Radiotherapy Biomaterials (iSRB)
Source: Pharmaceutics. 2023 Dec 14;15(12):2778. doi: 10.3390/pharmaceutics15122778 (PMC10747552; doi:10.3390/pharmaceutics15122778)
Supplement: Supplementary file 1 [file pharmaceutics-15-02778-s001.zip › Supplementary Table S7.pdf]

VRL – Maryland, LLC
401 Professional Drive, Suite 210
Gaithersburg, MD 20879
Phone: 1-800-804-3586

Date: 20 October 2023
Client: Johns Hopkins University
Pathologist: Dr. Dan Ragland

HISTOPATHOLOGY REPORT for Male Mice

Table S7.

Mouse Accession	23022781	23022782	23022783	23022784	23022785	23022786
Animal ID	No Treatment #1-D45	No Treatment #2-D45	No Treatment #3-D45	No Treatment #4-D45	No Treatment #5-D45	iSRB_Anti-CD40 #1-D45
LIVER	N			N		
**Microabscesses:** Mixed aggregates of inflammatory cells consisting primarily of neutrophils and fewer macrophages and/or lymphocytes, with one or more swollen and degenerating hepatocyes incorporated into the inflammatory focus.						2MF
**Microgranulomas:** Mixed aggregates of inflammatory cells consisting of up to 100 lymphocytes, macrophages, random neutrophils, and a small number of incorporated hepatocytes that are often swollen and or degenerate.			1MF		2MF	
**Lymphocytic aggregates:** Aggregates of purely lymphocytes, up to 100 cells, located in perivascular and parenchymal locations throughout the liver.		2MF				
KIDNEY: RIGHT	N	N	N	N	N	N
KIDNEY: LEFT	N	N	N	N	N	N
**Mouse Accession**	**23022787**	**23022788**	**23022789**	**23022790**		
**Animal ID**	**iSRB_Anti-CD40 #2-D45**	**iSRB_Anti-CD40 #3-D45**	**iSRB_Anti-CD40 #4-D45**	**iSRB_Anti-CD40 #5-D45**		
LIVER	N					
**Lymphocytic aggregates:** Collections of purely lymphocytes, up to 100 cells, located in perivascular and random parenchymal locations throghout the liver.						
**Microgranulomas:** Mixed aggregates of inflammatory cells consisting of up to 100 cells, of lymphocytes, macrophages, random neutrophils, and a small number of incorporated hepatocytes that are often swollen and or degenerate.		1MF	1MF	1MF		
**Microabscesses:** Mixed aggregates of inflammatory cells consisting primarily of neutrophils and fewer macrophages and/or lymphocytes, with one or more swollen and degenerating hepatocyes incorporated into the inflammatory focus.						
KIDNEY: RIGHT	N	N	N	N		
KIDNEY: LEFT	N	N		N		

**End Stage Kidney/hydronephrosis:** There is diffuse degeneration of renal tissue throughout the cortex, medulla, and pelvis. The pelvis is markedly dilated and is compressing the cortex to approximately half its original thickness. Renal tubules are diffusely dilated and contain intraluminal hyaline casts or necrotic tubular epithelial cells. The peripelvic suburothelium is expanded by lytic necrosis and infiltrates of neutrophils, lymphocytes, and cellular debris.			5D				

Scoring Definitions:

0= No finding **1= Minimal** **2= Mild** **3= Moderate** **4= Marked** **5= Severe**
N= Normal **M= Missing** **MF=Multifocal** **F=Focal** **D=Diffuse** **U=Unilateral**
B=Bilateral

Table S7. Pathology report corresponding to day forty-five post-treatment from harvested liver and kidney tissues.

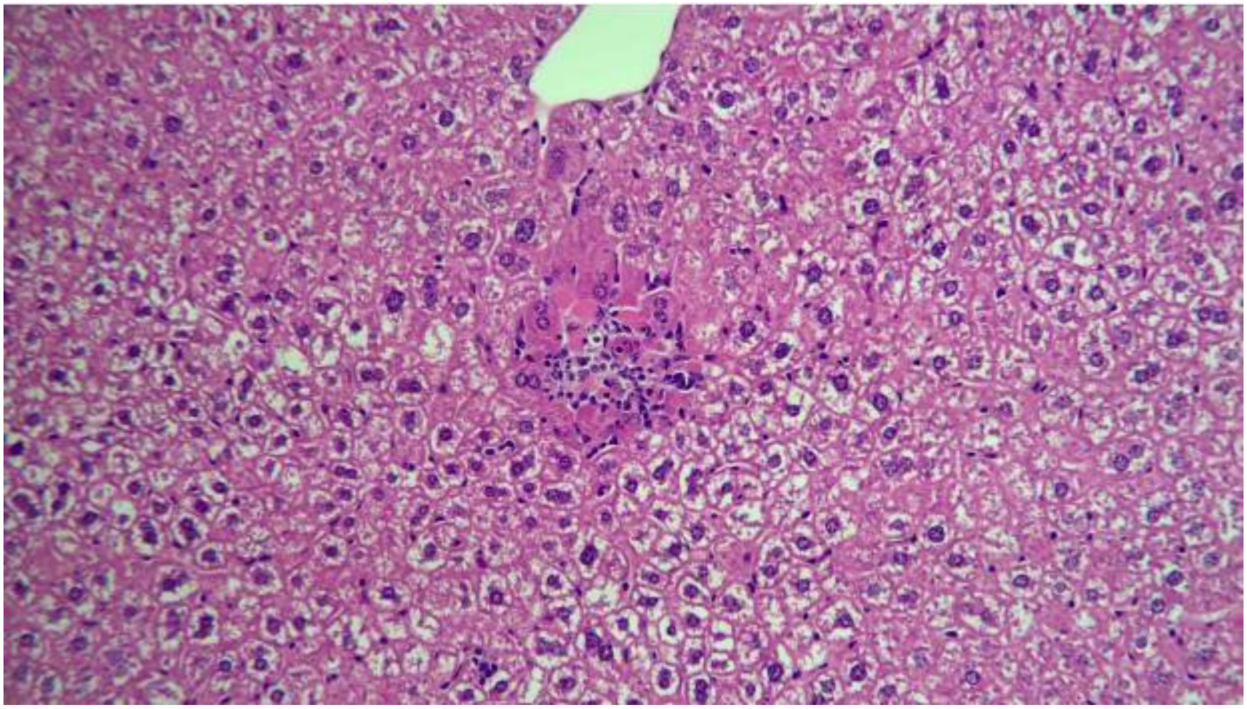

Mouse 23022783 liver showing representative microgranuloma. 200X

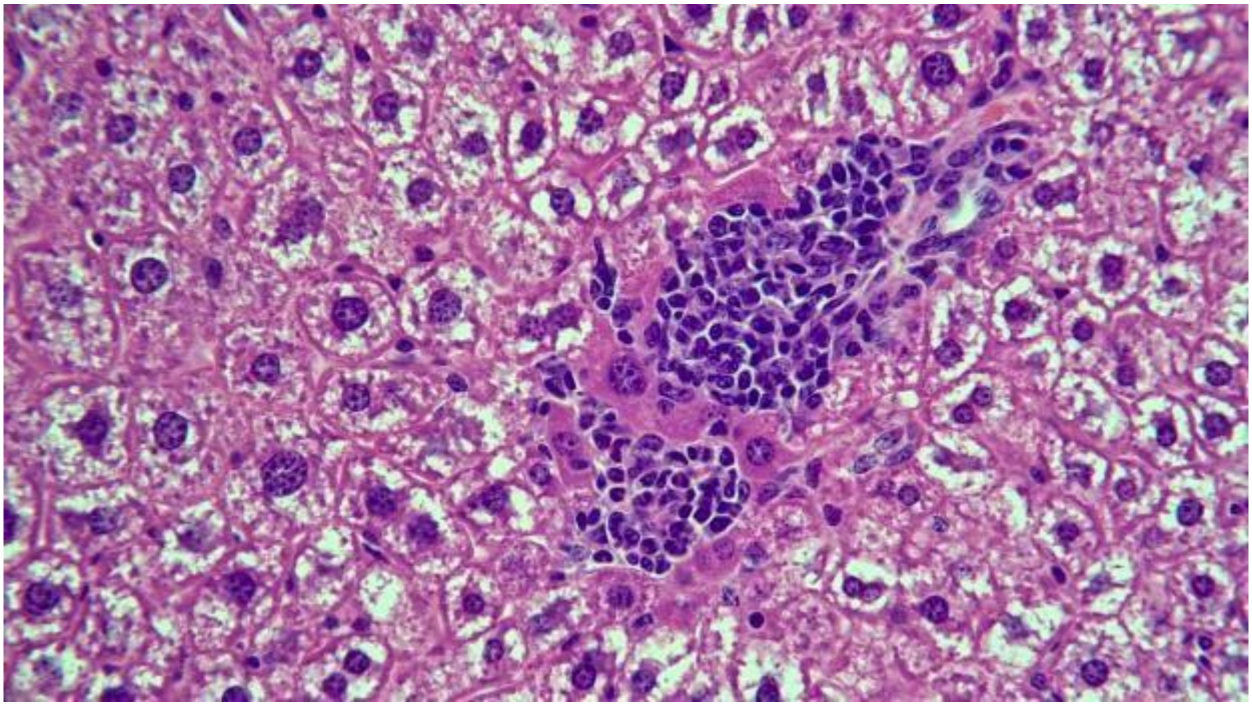

Mouse 23022785 liver showing representative microgranuloma. 400X

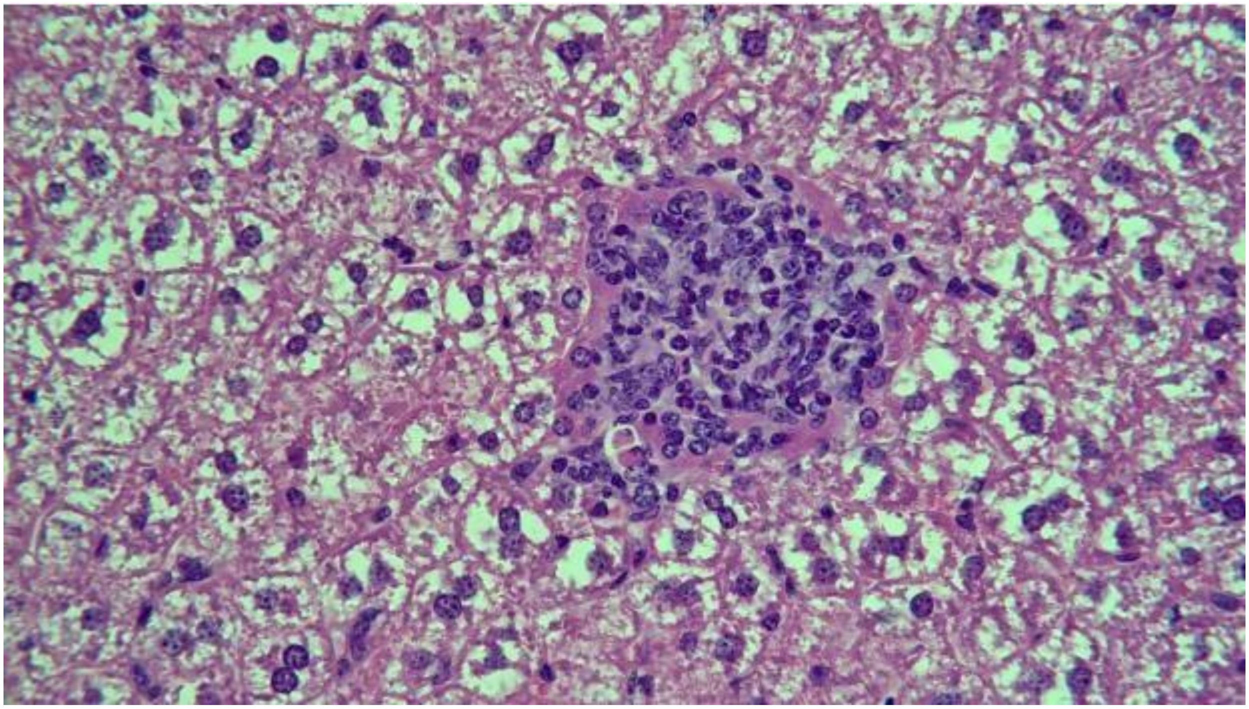

Mouse 23022786 liver showing representative microabscess. 400X

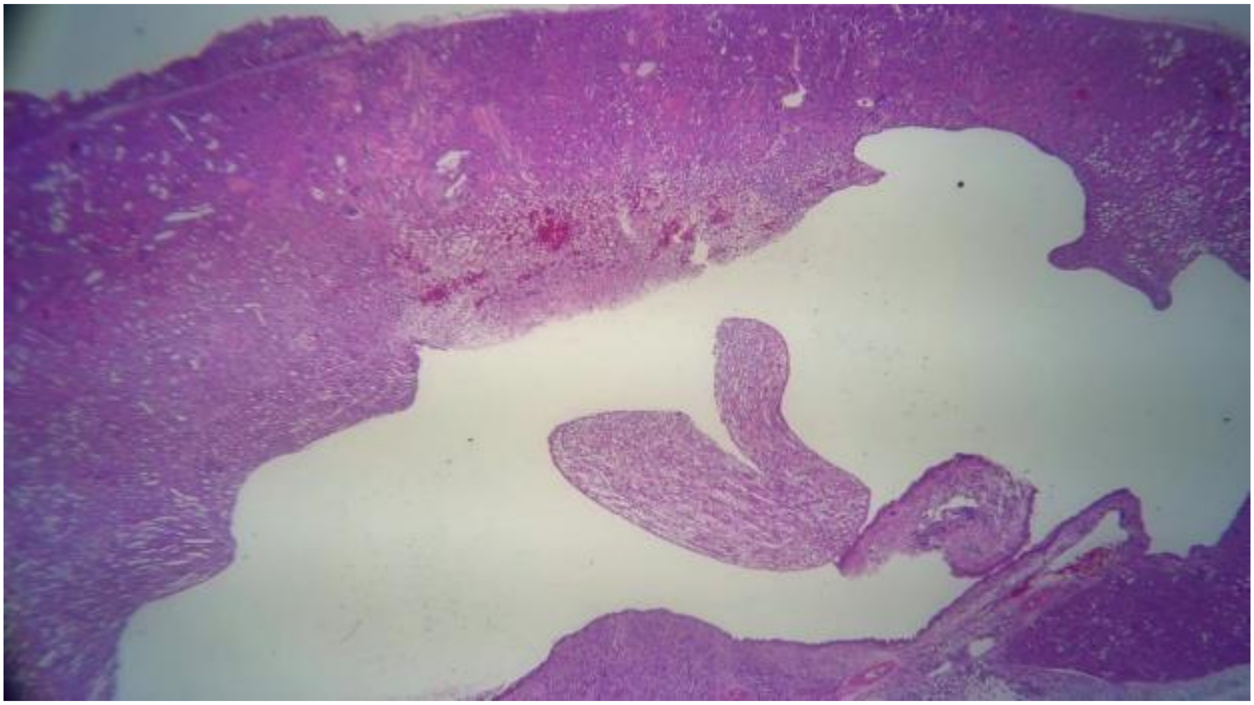

Mouse 23022789 kidney showing severe unilateral hydronephrosis and end stage degeneration. 20X
