# Supplementary figures and images for: Pre-Clinical Investigations of the Pharmacodynamics of Immunogenic Smart Radiotherapy Biomaterials (iSRB)
Source: Pharmaceutics. 2023 Dec 14;15(12):2778. doi: 10.3390/pharmaceutics15122778 (PMC10747552; doi:10.3390/pharmaceutics15122778)

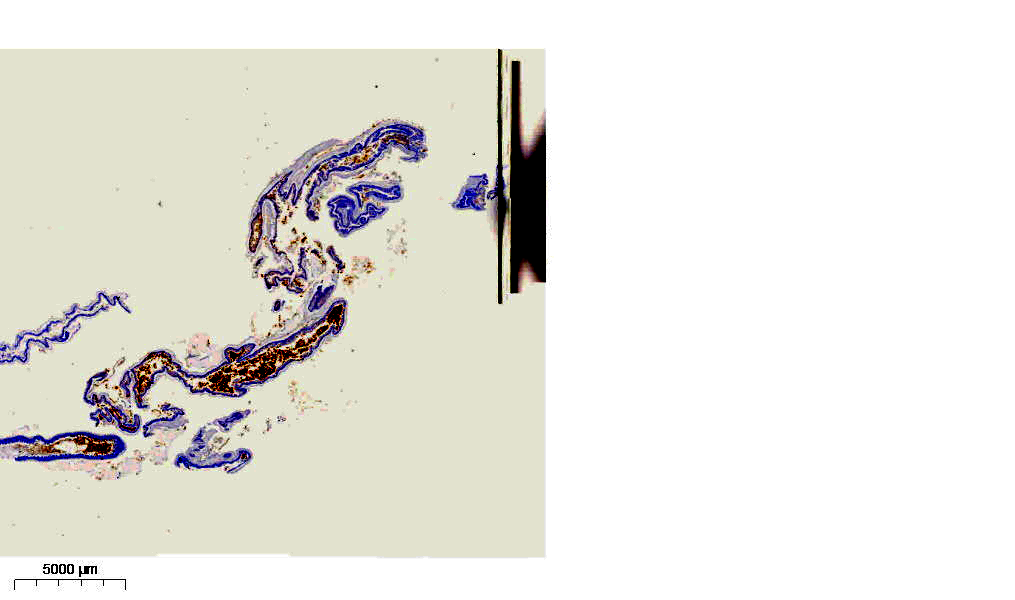

Supplement: Supplementary file 1 [file pharmaceutics-15-02778-s001.zip › Study4 Group1 LY6G GB11229 1-200.svs_0.3x.tif]

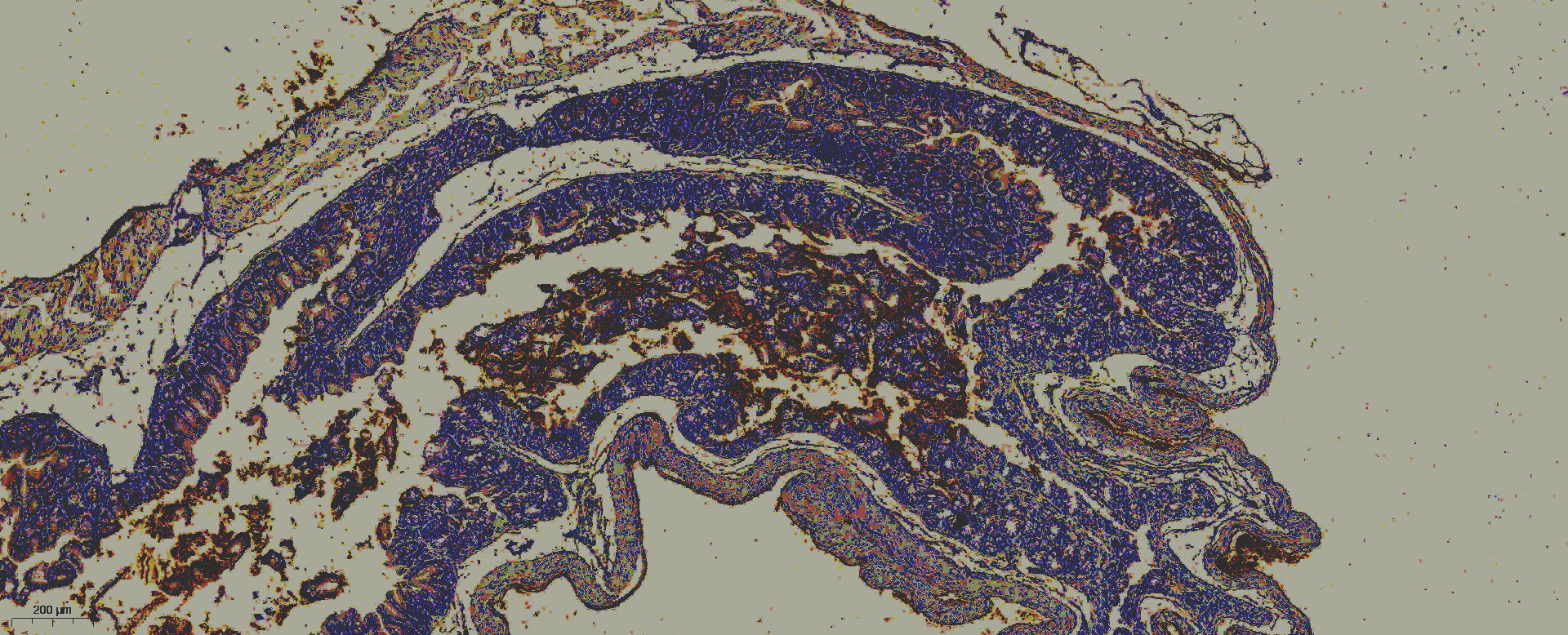

Supplement: Supplementary file 1 [file pharmaceutics-15-02778-s001.zip › Study4 Group1 LY6G GB11229 1-200.svs_10.0x.tif]

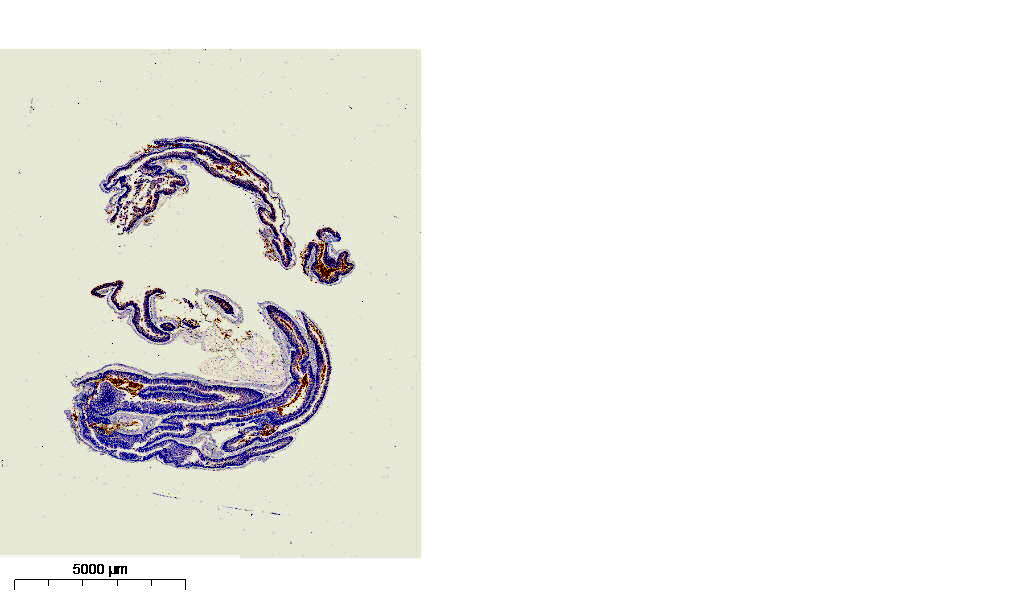

Supplement: Supplementary file 1 [file pharmaceutics-15-02778-s001.zip › Study4 Group3 LY6G GB11229 1-200.svs_0.4x.tif]

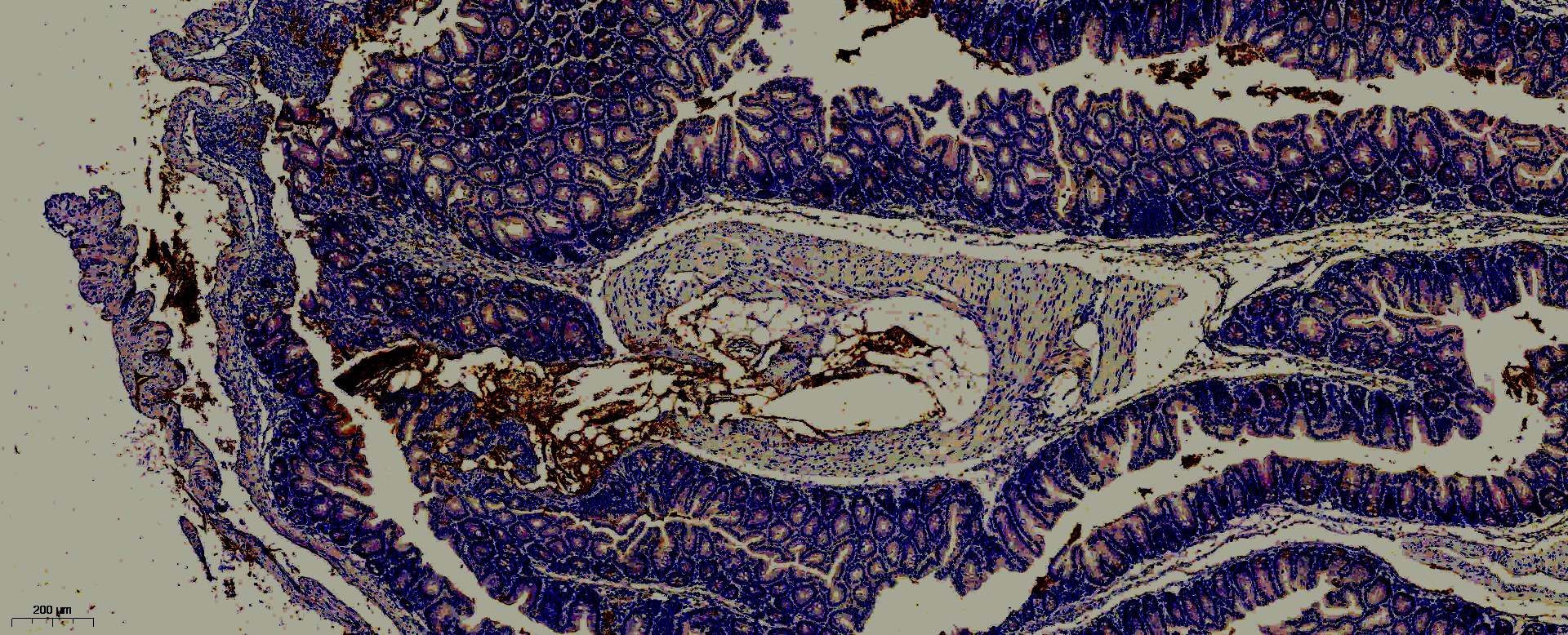

Supplement: Supplementary file 1 [file pharmaceutics-15-02778-s001.zip › Study4 Group3 LY6G GB11229 1-200.svs_5.0x.tif]

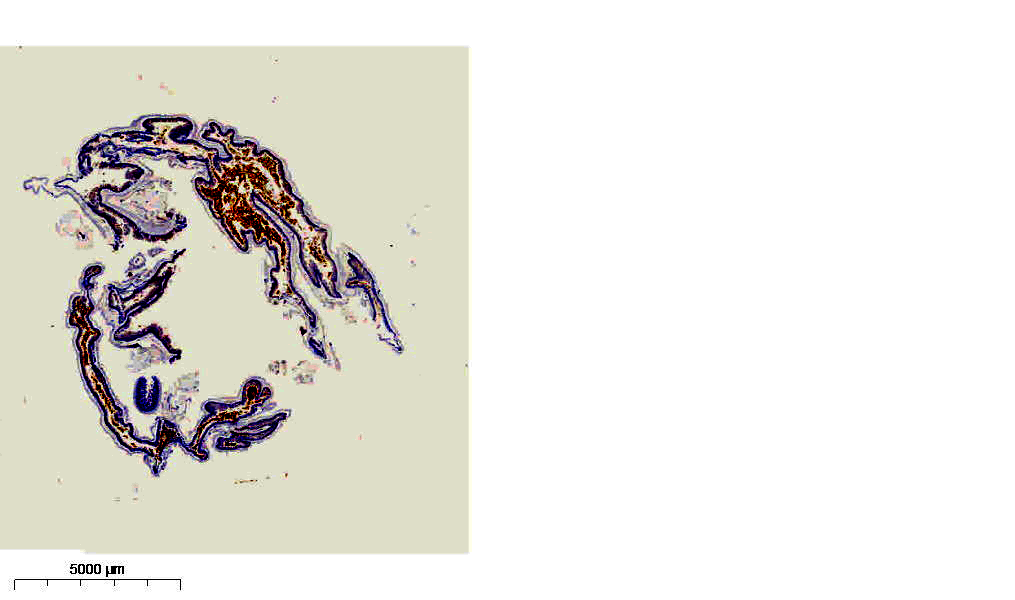

Supplement: Supplementary file 1 [file pharmaceutics-15-02778-s001.zip › Study4 Group4 LY6G GB11229 1-200.svs_0.4x.tif]

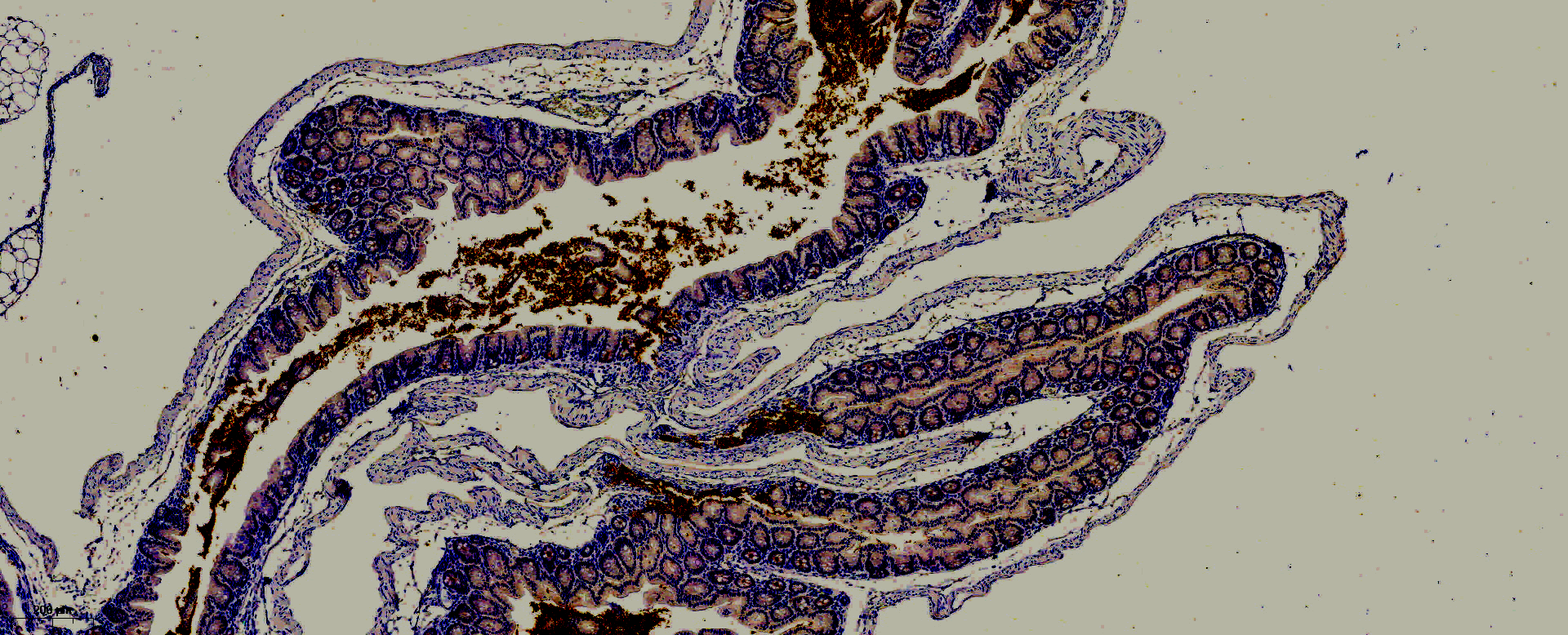

Supplement: Supplementary file 1 [file pharmaceutics-15-02778-s001.zip › Study4 Group4 LY6G GB11229 1-200.svs_5.0x.tif]
